# Investigating the Antigen Specificity of Multiple Sclerosis Central Nervous System-Derived Immunoglobulins

**DOI:** 10.3389/fimmu.2015.00600

**Published:** 2015-11-25

**Authors:** Simon N. Willis, Panos Stathopoulos, Anne Chastre, Shannon D. Compton, David A. Hafler, Kevin C. O’Connor

**Affiliations:** ^1^Department of Neurology, Yale School of Medicine, New Haven, CT, USA; ^2^Walter and Eliza Hall Institute of Medical Research, Parkville, VIC, Australia; ^3^Department of Medical Biology, University of Melbourne, Parkville, VIC, Australia; ^4^Department of Immunobiology, Yale School of Medicine, New Haven, CT, USA

**Keywords:** multiple sclerosis, B cell, autoantibody, autoantigen

## Abstract

The central nervous system (CNS) of patients with multiple sclerosis (MS) is the site where disease pathology is evident. Damaged CNS tissue is commonly associated with immune cell infiltration. This infiltrate often includes B cells that are found in multiple locations throughout the CNS, including the cerebrospinal fluid (CSF), parenchyma, and the meninges, frequently forming tertiary lymphoid structures in the latter. Several groups, including our own, have shown that B cells from distinct locations within the MS CNS are clonally related and display the characteristics of an antigen-driven response. However, the antigen(s) driving this response have yet to be conclusively defined. To explore the antigen specificity of the MS B cell response, we produced recombinant human immunoglobulin (rIgG) from a series of expanded B cell clones that we isolated from the CNS tissue of six MS brains. The specificity of these MS-derived rIgG and control rIgG derived from non-MS tissues was then examined using multiple methodologies that included testing individual candidate antigens, screening with high-throughput antigen arrays and evaluating binding to CNS-derived cell lines. We report that while several MS-derived rIgG recognized particular antigens, including neurofilament light and a protocadherin isoform, none were unique to MS, as non-MS-derived rIgG used as controls invariably displayed similar binding specificities. We conclude that while MS CNS resident B cells display the characteristics of an antigen-driven B cell response, the antigen(s) driving this response remain at large.

## Introduction

Multiple sclerosis (MS) is the most common neurological disease affecting young adults. MS is an inflammatory disease of the central nervous system (CNS) characterized by immune cell infiltration and demyelination of the brain and spinal cord that leads to physical disability (1). Although the cause of the demyelination is not entirely clear, many studies have implicated T cells as the dominant immune cell type contributing to disease pathology. However, growing evidence also suggests that B cells play an active role in the disease (2). A recent ENCODE study (3) implicated B cells second only to T cells among the cell types most affected by MS susceptibility genes. B cells are found at sites of tissue injury in the CNS. They are also found in the CSF, white matter lesions, gray matter, and in the meninges, where they form lymphoid-like tissue aggregates (4) that associate with proximal tissue damage (5). Furthermore, they are responsible for the production of the oligoclonal immunoglobulin bands (OCB) in the spinal fluid that are a hallmark of the disease. Their roles as both effective antigen-presenting cells (6) and immune response regulators (7) have recently been appreciated. Finally, B cell depletion, which has emerged as a beneficial therapeutic approach for MS, confirms that B cells contribute to MS pathology (8).

A number of autoimmune demyelinating diseases of the CNS are associated with a robust B cell response, and in several cases, antigens implicated in this response have been identified. Neuromyelitis optica (NMO) serves as a prototypical example of demyelinating CNS autoimmunity associated with B cells. Most NMO patients produce antibodies [both serum immunoglobulin (9) and CSF-derived IgG (10)] that bind the water channel aquaporin-4 (AQP4). These antibodies have been shown to be derived in part from a clonally expanded B cell pool located within the CSF (11, 12). Other examples of B cell-related autoimmune demyelinating CNS conditions are pediatric MS and acute disseminated encephalomyelitis (ADEM), where antibodies to myelin oligodendrocyte glycoprotein (MOG) have been identified (13, 14).

During subacute and chronic active infections of the CNS such as Lyme neuroborreliosis or subacute sclerosing panencephalitis (SSPE), OCB are found in the CSF and resolve when the infection is cleared. In SSPE, brain-derived, recombinant immunoglobulin can be specifically absorbed by the causative virus, namely, the measles virus (15). The humoral immune response in MS shares many similarities with that seen in SSPE, NMO, and other inflammatory diseases of known cause. The MS CSF often includes elevated immunoglobulin levels and OCB, both of which are derived from B cells residing in the CSF and CNS tissue (16, 17). The CNS B cells in SSPE, NMO, and MS display the characteristics of an antigen-driven response, with high levels of clonal expansion and somatic hypermutation in IgG variable regions, all of which are consistent with post-germinal center activation (12, 18–20). However, in contrast to SSPE and many infectious encephalopathies, the antigen target of the CNS-associated immunoglobulin is not known in MS. Given these similarities and the clear evidence for an antigen-driven response displayed by MS CNS resident B cells, the identification of the autoantibody targets in MS is of substantial interest.

The search for specific autoantibodies in MS has been an area of focus for decades, but the antigens targeted by MS autoantibodies have remained elusive. Many studies have focused on serum antibodies given their accessibility and that serum autoantibodies have been identified in several diseases. Myelin basic protein (MBP) autoantibodies are detected in a very small subset of MS patients (21). MOG autoantibodies appear to be reliably found in a small subset of patients with MS (14) that are primarily pediatric. More exhaustive lists of candidate MS antigens can be found in a number of valuable reviews (22, 23). Numerous candidate autoantibody targets have been reported [reviewed in Ref. (2, 24, 25)], but none have met all the criteria that would allow for widespread acceptance as a genuine disease-associated MS autoantibody. These criteria would, at the very least, include such characteristics as disease specificity, reproducible detection among different laboratories, and different patient cohorts and disease relevance in terms of diagnosis, prognosis, or contribution to immunopathology. Newly identified candidate antigens of interest include contactin-2 (26), ATP-sensitive inward rectifying potassium channel KIR4.1 (27), and sperm-associated antigen 16 (28), all of which are undergoing validation. Although a number of serum-derived antibody targets, such as MOG, can be found in small subsets of MS patients, most of those identified in serum have failed to be sensitive and specific markers for the disease. Some candidate autoantigens appear to be enriched in (29) or restricted to the CSF relative to serum, such as recombination signal binding protein for immunoglobulin kappa J region (RBPJ) (30). These autoantigens also represent a small subset of patients that have not yet defined a unique clinical phenotype.

To date, no antigen has emerged as a validated and widely accepted “MS antigen.” We reasoned that the recombinant IgG (rIgG) derived from the clonally expanded and antigen experienced B cells that populate sites of tissue damage in the MS CNS are likely to represent the most enriched sources of disease-relevant antibody. Accordingly, we sought to explore the specificity of such MS CNS-derived immunoglobulin. To this end, we produced rIgG from a series of clonally expanded CNS-derived B cells from different MS CNS specimens and controls. The rIgGs were then screened against previously implicated candidate antigens as well as with high throughput approaches that multiplex large sets of antigens such as whole protein arrays and CNS-derived cell lines. In all of the screening approaches, an effort was made to maximize preservation of conformational and post-translational epitopes. This study, to our knowledge, represents the first time that such a technically demanding approach utilizing recombinant antibodies from CNS lesion-derived B-cells has been employed toward antigen discovery in MS.

## Materials and Methods

### Ethics Statement

Patient-derived specimens did not include personally identifiable private information or intervention or interaction with an individual and were accordingly collected under an exempt protocol approved by the Human Research Protection Program at Yale School of Medicine.

### Subject Specimens

Tissues were dissected at autopsy from six subjects with clinically defined MS. Five of the six subjects had a progressive clinical course and one had a relapsing remitting clinical course. Collected tissues included lesions and meningeal follicles. Our group previously reported the characteristics of the B cells that infiltrated these specimens (18, 31). Control tissues, which harbored robust B cell infiltrates, included germ cell tumors and muscle tissue from patients with inclusion body myositis (IBM), both of which have been previously described by our group (32, 33).

### Laser Capture Microdissection and B Cell Variable Region Cloning

Central nervous system tissue was sectioned at 12 μm on a microtome/cryostat, mounted onto a glass slide then fixed in 75% ethanol for 30 s. For the identification and capture of individual B lineage cells, the tissue was stained with mouse anti-CD20 or anti-CD38 antibodies (Accurate Chemical & Scientific) after fixation, then counterstained with poly-horseradish peroxidase anti-mouse IgG (Ivax Diagnostics). The tissue was then dehydrated in consecutive washes of 75, 95, and 100% ethanol then xylene. Cells were captured with a PixCell IIe laser capture microdissection instrument and CapSure Macro caps (Arcturus) and immediately stored at −80°C. RNA was isolated with the Absolutely RNA Nanoprep Kit (Stratagene) according to the manufacturer’s protocol. B cell variable regions were cloned and analyzed according to procedures that we have previously reported (18, 33).

### Recombinant IgG Synthesis and Purification

Multiplex PCR was used to amplify the immunoglobulin variable heavy chain (VH) and variable light chain regions (VL). These products were subsequently directionally sub-cloned behind the CMV promoter into a pcDNA3.3- or pCEP4-based vector constructed in-house to harbor the human immunoglobulin IgG_1_ heavy chain and kappa constant domains, respectively. The heavy chain vector was modified to contain a C-terminal affinity tag (HA-hemagglutinin). Expression and purification of the recombinant whole human IgG was performed with protocols that we have previously described (34). Recombinant IgGs (rIgG) were prepared from the matched variable heavy (VH) and light regions (VL) derived from either laser captured single cells or by matching the most highly expressed VH and VL clones from each library.

### Solid Phase Immunosorbent Assays

Solid phase ELISA was performed to evaluate rIgG recognition to a number of candidate antigens. These assays were performed using an approach that we have previously described (21). Similarly, the DELFIA assay for the detection of antibody binding to MBP was performed using an approach that we have previously described (35).

### ProtoArray

ProtoArray Human Protein Microarrays version 5.0 (Life Technologies), containing approximately 9,400 unique full-length human proteins, were used. The assay was performed according to the manufacturer’s instructions as we have previously described (30). Briefly, protein microarray slides were probed with rIgG pools (normalized for total IgG content) by overnight incubation at 4°C. Bound rIgG was detected with an Alexa Fluor 647-conjugated goat anti-human IgG (Life Technologies). The arrays were then scanned using a GenePix 4200A (Molecular Devices) fluorescent microarray scanner and analyzed with GenePix software. The standard score (*Z*-score) for binding to each antigen was determined using the Immune Response Profiling function within Prospector software (Life Technologies). The selection criteria applied for binding to be considered positive was a *Z*-score >3.

### Cell-Based Antibody Binding Assays

The cell-based assay for MOG binding was performed with Jurkat cells that were transfected to express a fusion protein that included the extracellular domain of human MOG linked to GFP. Antibody binding was then measured using an approach as we have previously described (13).

Cell lines were prepared for surface binding screening using methods we have previously described (34). Briefly, cells were incubated with each recombinant antibody at a concentration of 5 µg/ml, and then incubated with a polyclonal goat anti-human IgG AlexaFluor 488-labeled antibody (Life Technologies) to detect binding. Cells were resuspended in BD Cytofix (BD Biosciences) and stored at 4°C in the dark until being analyzed by flow cytometry with a FACSCalibur flow cytometer (BD Biosciences). Median fluorescence intensity (MFI) was used to assess binding of MS-derived and control rIgG to the CNS and control cell lines. Similarly, intracellular staining was performed in the same manner as that described for surface binding except for the addition of the permeabilization step, which was facilitated using Cytofix/Cytoperm (BD Biosciences) according to the manufacturer’s instructions.

## Results

### Generation of Recombinant IgGs from MS Brain

To explore the specificity of the antibodies produced by CNS-derived B cells, we prepared rIgG from immunoglobulin variable region sequences derived from MS and control tissues. The MS cohort included rIgG constructed from MS autopsy tissue specimens from six subjects, MS-A thru MS-F (Table 1). Five of the six subjects were female and one male, with ages ranging from 34 to 65 years at the time of death. Five of the MS subjects had a progressive course, while one had relapse-remitting MS. Disease duration ranged from 2 to 20 years. Six recombinant antibodies (Table S1 in Supplementary Material) were derived from clones present in MS-A; four recombinant antibodies each were derived from clones present in MS-C and MS-D and a single recombinant antibody was derived from each of the MS-B, MS-E, and MS-F tissues. All of the MS-derived rIgGs were constructed from clonally expanded cells that displayed evidence of affinity maturation including class switching and the accumulation of somatic mutations (Table S2 in Supplementary Material). The control rIgGs were derived (Table 1) from either an intracranial germinoma (GCT-A) or muscle tissue from two different patients with inclusion body myositis (IBM) (IBM-A and IBM-B). We previously demonstrated that the B lineage cells infiltrating both the tumor and muscle tissue (32, 33) shared antigen-driven characteristics that were similar to those representing the MS cohort (Table S2 in Supplementary Material). Specifically they had class switched to IgG, had accumulated somatic mutations, and were remarkably clonally expanded into families that included numerous clonal variants. Nine recombinant antibodies (Table S1 in Supplementary Material) were derived from clones present in the germinoma (GCT-A1 thru GCT-A9) and three each from the two IBM specimens (IBM-A1-3 and IBM-B1-3). Finally, a well-described (36) monoclonal antibody that recognizes MOG, which we humanized, was included in the control cohort (h8-18C5) (Table S1 in Supplementary Material).

**Table 1:** **Subject demographics and source of MS and control tissue**.

Case	Age (years)	Gender	Clinical course	Disease duration (years)	Source
MS-A	43	F	Progressive MS	20	Autopsy
MS-B	34	F	Progressive MS	2	Autopsy
MS-C	39	F	Progressive MS	13	Autopsy
MS-D	38	F	Relapsing remitting MS	n.a.	Autopsy
MS-E	65	M	Progressive MS	n.a.	Autopsy
MS-F	49	F	Progressive MS	14	Autopsy
GCT	<18	M	Intracranial germinoma	n.a.	Resection
IBM-A	>40	M	Inclusion body myositis	n.a.	Biopsy
IBM-B	>40	M	Inclusion body myositis	n.a.	Biopsy

*n.a., data not available*.

### Screening MS rIgG for Binding to Candidate Antigens

**Figure 1:**
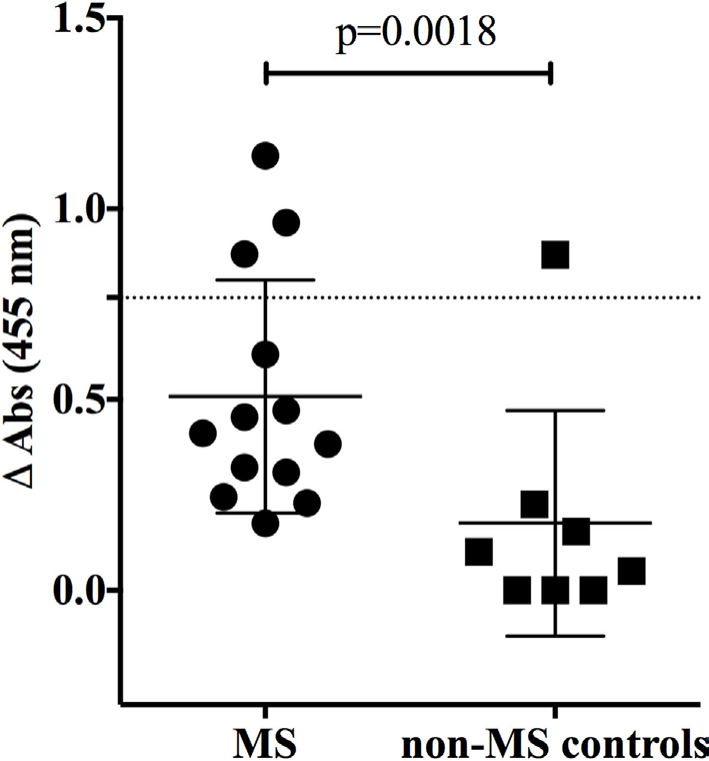
**MS and control-derived rIgG binding to neurofilament light (NF-L) by solid phase ELISA**. MS-derived rIgG (*n* = 13) and control rIgG (*n* = 8) derived from a germinoma were tested by solid phase ELISA for binding to NF-L. The specific samples included in the assay are shown in the Supplementary Material. Each dot or square represents the binding of a single rIgG. The dashed line indicates the mean +2 SD of the control germinoma-derived cohort (0.76). Values above this line were determined to be positive (95% CI). To correct for non-specific binding, the reported ELISA signal (ΔOD) was calculated by subtracting the signal generated by binding to glyceraldehyde 3-phosphate dehydrogenase (GADPH) from that of the NF-L. The mean and SD are shown for each data set. Statistical differences are indicated when significant. Data associated with each rIgG for the MS and control groups are shown in the Supplementary Material.

To investigate the specificity of CNS-derived antibodies, we began by screening against candidate antigens that have previously been implicated in MS. A DELFIA and an ELISA assay were performed to test MBP (37) and contactin (26), respectively. Differences in binding to MBP and contactin between the MS and control rIgG were unremarkable (*not shown*). We also used an ELISA assay to assess binding of MS and control rIgG to the intracellular protein neurofilament light (NF-L) (38). Ten MS-derived antibodies that were tested showed modest binding to NF-L (Figure 1) while three antibodies (MS-B1, MS-C2, and MS-C4) displayed strong binding with absorbance values that exceeded the mean +2SD of the control data set (*benchmark for strong positive binding*). The difference between the MS and the control group was significant (*p* = 0.0018, *Mann–Whitney test*). However, binding was not restricted to MS-derived antibodies as a germinoma-derived antibody (GCT-A6) was also positive, indicating a lack of specificity for MS in the rIgG cohorts.

**Figure 2:**
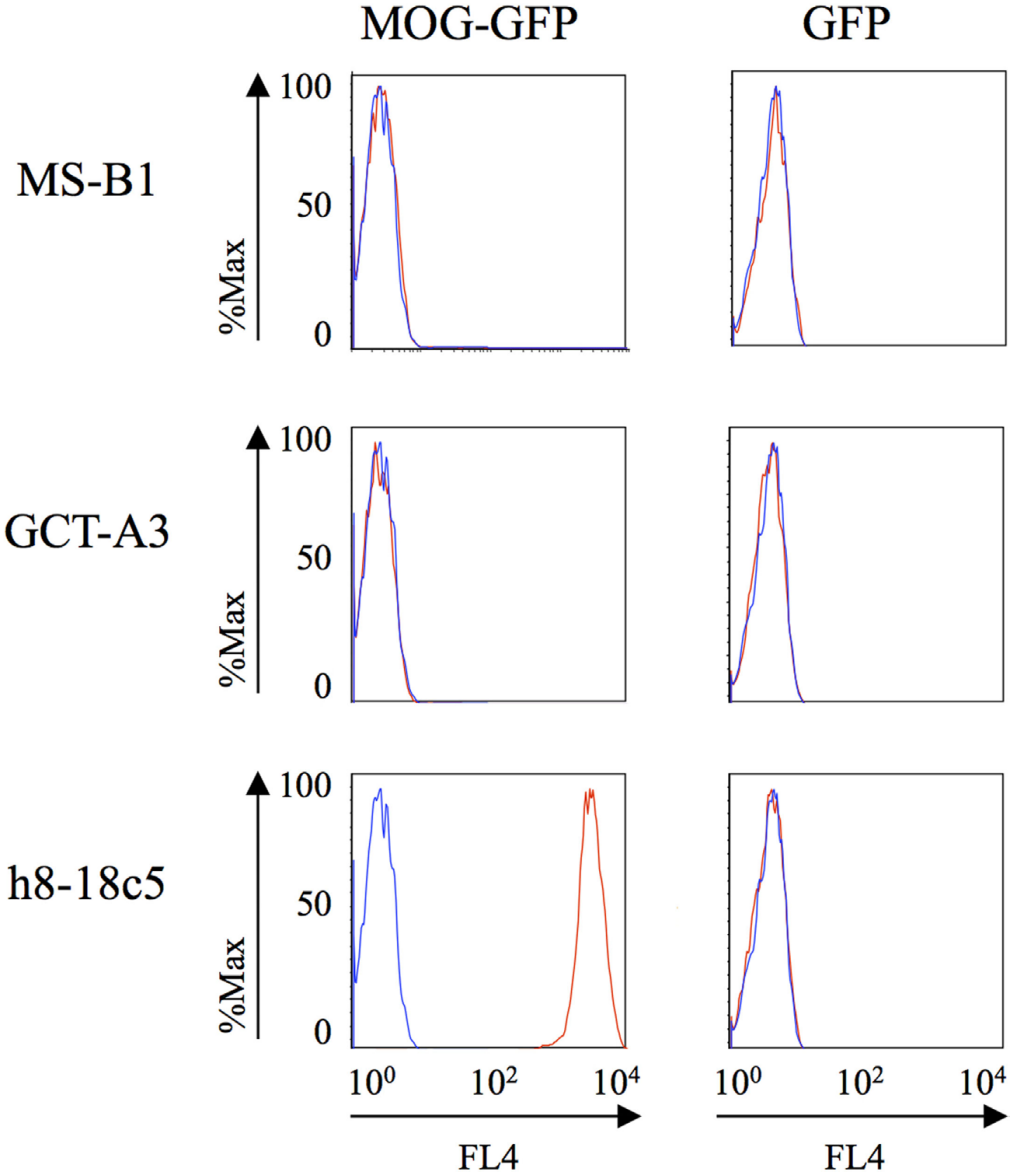
**MS and control-derived rIgG binding to MOG detected with a cell-based assay**. Representative binding of MS (MS-B1) or germinoma control-derived (GCT-A3) rIgG to Jurkat cells transfected with MOG-GFP (left column) or GFP alone (right column). Histograms show the MFI of transfected cells gated on those that were positive for both GFP and a florescent anti-human secondary antibody (red). The blue histograms show secondary antibody alone. A humanized monoclonal antibody, h8-18c5, specific for human MOG served as a positive control for the Jurkat–MOG–GFP binding. FACS data for additional rIgGs from the MS and control groups are shown in the Supplementary Material.

We also examined binding to MOG; autoantibodies to MOG have recently been described in a small subset of MS patients (13), in pediatric MS (14) and in NMO (39). MOG binding was evaluated using a cell-based assay that preserves conformational epitopes and, accordingly, has become a widely accepted approach for detection of such antibodies (13). Robust binding by the humanized monoclonal anti-MOG monoclonal antibody (h8-18C5) was recorded (Figure 2). The clear recognition of MOG by our humanized h8-18C5 demonstrates that our recombinant expression system produces fully functional whole human IgG and did not introduce any artifacts that might confound native specificity. Applying this approach to the MS- and germinoma-derived rIgG demonstrated that none of these antibodies recognized MOG expressed on the surface of the cells (Figure 2; Figure S1 in Supplementary Material).

### Screening MS rIgG Reactivity with High-Throughput Protein Arrays

**Figure 3:**
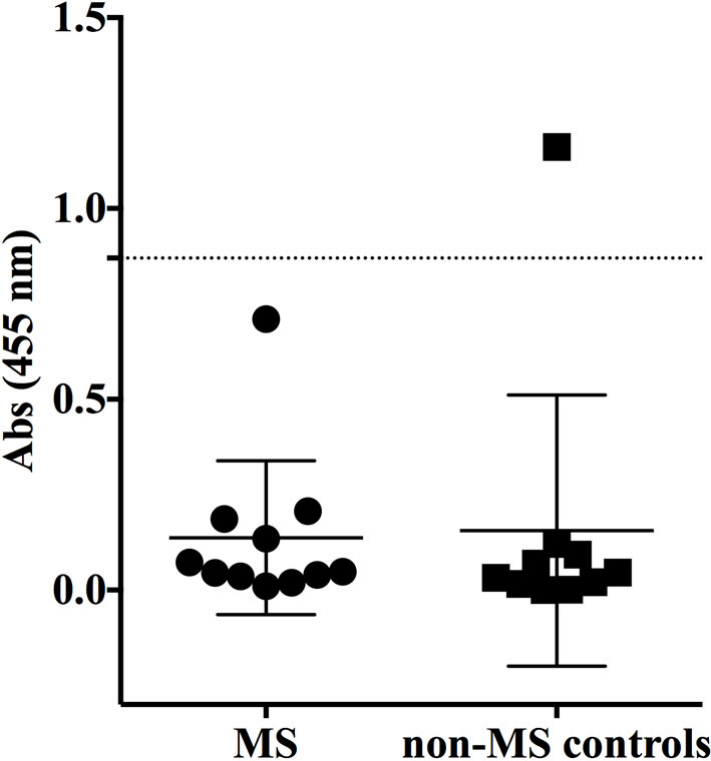
**MS and control-derived rIgG binding to protocadherin gamma (PCDHGC3) by solid phase ELISA**. MS-derived rIgG (*n* = 11) and control rIgG (*n* = 10) derived from a germinoma and muscle tissue were tested by solid phase ELISA for binding to protocadherin. The specific samples included in the assay are shown in the Supplementary Material. Each dot or square represents the binding of a single rIgG. The dashed line indicates the mean +2 SD of the control-derived cohort (0.87). Values above this line were determined to be positive (95% CI). The mean and SD are shown for each data set. Statistical differences are indicated when significant. Data associated with each rIgG for the MS and control groups are shown in the Supplementary Material.

Having shown no specificity for the MS-derived rIgG to several candidate antigens, we sought to expand the search by using an unbiased library of antigens that could be screened in a high-throughput manner. To this end, we examined the rIgG specificity from the MS and control cohorts with a commercially available protein array composed of approximately 9,400 unique full-length human proteins that were expressed in a system such that the products included some physiologic post-translational modifications and processing. The rIgGs from both the MS and controls groups were pooled so that three rIgG were included on each array during the initial scouting to maximize efficient use of the arrays. A total of three MS and three control arrays were run. Target antigens that were identified by at least one MS antibody pool that did not react with any of the control groups are shown in Figure S2 in Supplementary Material. In most instances, antigen targets were found on a single MS array; however, several were found on two of the three MS arrays. Of these, protocadherin gamma subfamily C, three (PCDHGC3), transcript variant three was of particular interest as a candidate autoantigen as protocadherin isoforms, include extracellular domains, are predominantly expressed in the nervous system and have been implicated in human neurological disorders (40, 41). Given their attractive role as candidate MS antigens we investigated this specificity further. To do this we tested binding to PCDHGC3 protein by ELISA with individual rIgGs rather than pooled mixtures (Figure 3). The MS-derived rIgG MS-C2 that was present in the pool that bound PCDHGC3 on the ProtoArray (MS array 2) bound to the protein. However, binding was not observed for the individual rIgGs present on the second array that also identified this target (MS array 3). Furthermore, the difference between the MS and the control group was not significant (*p* = 0.3432, *Mann–Whitney test*) and binding was not restricted to MS-derived antibodies as a germinoma-derived antibody (GCT-A10) also was positive in the ELISA, indicating a lack of specificity for MS in the rIgG cohorts.

### Screening MS rIgG for Binding to Human CNS-Derived Cell Lines

Limitations of the ProtoArray for autoantigen discovery include the underrepresentation of membrane proteins on the array that would have an extracellular domain accessible to antibody and the possibility of altered structural conformation. Cell-based assays can circumvent such restraints. Increased binding to extracellular components of oligodendrocyte precursor and neuronal-derived cell lines by MS serum immunoglobulin compared to healthy controls has been reported (42). Thus, to complement the array data and address its limitations we screened the MS and control rIgG by flow cytometry for binding to extracellular antigens present on the surface of the CNS-derived cell lines including a human oligogendroglioma cell line (HOG) and a human neuroblastoma cell line (SKNSH). Applying this approach, we found that none of the MS or control rIgG tested bound to the surface of either of the CNS cell lines (Figure S1 in Supplementary Material).

We were also interested to test whether the rIgGs would recognize antigens that reside in the cell cytoplasm as CNS resident antibodies may be exposed to such antigens during tissue damage. Flow cytometry was used to screen for intracellular binding of the rIgGs to the HOG cell line. All of the rIgGs from the MS and control cohorts bound to the permeabilized cells (Figure S3 in Supplementary Material). A similar pattern of binding was obtained with the 293T cell line (*not shown*). Overall, there were no significant or remarkable distinctions between the flow cytometry MFI histograms of the two cell lines for both the MS and control rIgGs, indicating that intracellular components are frequent non-specific targets of antibodies.

## Discussion

The purpose of our work was to investigate the antigen specificity of the humoral immune response in MS. We focused on those B cells that reside at the site of tissue damage in MS brain as this compartment likely represents an enrichment of disease-associated antibodies compared to serum and to the CSF. Two studies have leveraged a similar approach to examine the specificity of antibody-secreting cells present in the MS CSF (43, 44). While these studies suggested that MS-derived B cells recognize myelin, also derived from MS brain, the specific myelin component could not be identified. Moreover, no reactivity was observed to the major myelin protein antigens, MBP, and MOG. Using CNS tissue-derived B cell products, we found that MS-derived antibodies did recognize a number of candidate antigens, but when challenged with a matched set of appropriate controls the MS specificity did not persist. We carefully selected control rIgG that shared the same properties as the MS-derived rIgG. In studies outside of our MS program, we have characterized the B cells that infiltrate particular solid CNS tumors and the muscle tissue of patients with myositis. In both instances, similar to what we observed in MS, the tissue-enriched B cell repertoire was class switched, clonally expanded and somatically hypermutated. Clones were selected from these repertoires in the same manner as those selected from our MS cohort. In light of these considerations, we suggest that the set of controls we employed are superior to controls produced by naïve B cells or random memory B cells from the circulation. In spite of using candidate antigens coupled with systems biology approaches, we did not identify a validated target for the B cells that reside in the MS CNS tissue. Overall our study highlights the difficulty inherent in antigen discovery approaches, but provides a methodological road map for improvement in the field with emerging technology. We postulate that the target of MS CNS B cells may be: an undiscovered common antigen that may include post-translational modification; antigen(s) possibly exposed through tissue damage; a collection of target antigens that vary among smaller MS population subsets and/or vary based on compartments (serum/CSF) or an infectious agent in the brain itself that would not be identified using our current screening approaches.

Antibody-independent mechanisms may help to explain the pathological contribution of B cells to MS. However, the antigen-driven characteristics of MS CNS B cells still point toward a role for antibody-dependent mechanisms. It is intriguing that B cells have been shown to form structures that resemble ectopic germinal centers in the meninges of MS patients (5, 18, 45) where they display all of the characteristics of an antigen-driven response. Similar organized structures have also been found in a number of autoimmune disease tissues (46) and often in different solid tumors (33). As an example, tertiary lymphoid structures have been identified in the thymus tissue of myasthenia gravis (MG) patients (47), where they have been shown to contribute to MG autoantibody production. With respect to MS, the question remains as to the target of the B cell response. Are the B cells directed toward a CNS target that is involved in disease initiation or is the B cell response generated as an indiscriminate secondary response to the dead tissue, rather than the cause of the pathology in the first place? In the case of organized tumor immune cell infiltrates, it is expected that there is not an underlying immune dysregulation that would be expected in autoimmune conditions. Given the similarity between these organized infiltrates, this leaves the possibility open that the process in MS may not entirely be a product of abnormal immune regulation. Are the antibodies in the CNS a normal immune response to the ongoing tissue damage that occurs? This possibility could provide an explanation for the B cell response in MS CNS tissue that it is part of apoptotic cell recognition, secondary to the disease pathology and part of a normal response.

Antigen discovery efforts are not without limitations and ours in not an exception. First, we employed LCM to confirm VH and VL domains were endogenously paired. However, this approach provides a low yield of paired VH and VL domains from single cells derived from autopsy tissue, so we chose to also pair domains based on their representation in the respective libraries. We acknowledge that pairing of VH and VL domains based on their dominant distribution in the repertoire is not a guarantee that they were naturally paired in a single cell; however, it represents the best possible means toward obtaining rIgG from autopsy tissue with current technology. High-throughput technology to pair heavy and light chains from single cells (48) was not available when our study began and this technology is currently limited to use with cells in suspension, which is not possible with cryopreserved autopsy tissue. Pairing based on the most highly represented VH and VL domains was successfully demonstrated in a vaccination setting (49). Furthermore, “knock in” transgenic mice, which express the VH from the 8-18C5 anti-MOG antibody, produce antibody using the endogenous light chain repertoire of the host, and a large fraction of the antibodies recognize MOG regardless of the light pairing, demonstrating the dominant contribution of the VH in target binding (50).

The second limitation concerns the antigen sources. Although our screening strategy was thorough, it was not exhaustive. We focused on proteins since they constitute antigenic targets more frequently than other molecules. In testing individual antigens, we employed ELISA, which allows for rapid testing of multiple samples, and cell-based assays where physiological epitopes are better emulated. Our use of cell lines offers the advantage of presenting multiple cell surface candidate antigens that are present in the CNS. However, low or sparse endogenous expressional levels of proteins on the cell surface or altered expression can affect antibody binding (51) to such cells. Using tissue offers deliberate sourcing of compartments but also can present technical challenges including antigen recovery in fixed tissue used in immunohistochemistry and low antigen abundance when performing western blots or immunoprecipitations.

The protein array, we employed, offers the advantage of highly enriching low abundance proteins that might not be detectable when presented in other formats such as tissue. Furthermore, inclusion of whole proteins offers the potential for increased sensitivity and specificity compared to peptide arrays and phage display libraries. However, the arrays that we used include some shortcomings, such as an abundance of intracellular proteins and an absence of comprehensive post-translational modifications. Moreover, certain surface proteins that have been implicated in demyelination such as contactin-1 and 2, contactin-associated protein-1 and neurofascin-155 were absent from the panel. Our array results were negative for several surface proteins (catenins, integrins, tetraspanins, claudins) that could be considered biologically plausible targets (52) and were also negative for myelin-associated proteins, as well as for the previously identified intracellular targets of CSF antibodies, myelin-associated enzyme CNPase and RBPJ (30, 53, 54). Positive array hits primarily included several intracellular proteins. Abundant, intracellular protein autoantigens may be useful biomarkers only after extensive validation and scrutiny. Our cell-based assays comparing extracellular and intracellular binding clearly illustrate lack of specificity of the latter. Second, intracellular proteins can be present in many cell types, and therefore lack the tissue specificity that is often associated with validated autoantigens. Thirdly, the question of access to circulating antibodies to intracellular proteins cannot be easily answered. Overall, both our data and other whole protein array studies suggest that IgG specificity may vary among subsets of MS patients (30, 55, 56).

## Conclusion

A comprehensive method to systematically characterize and screen disease-associated immunoglobulins is needed. Such an ideal system is not yet available, but as one is developed it should include biologically relevant whole proteins presented in their native biological state, that is, with endogenous post-translational modifications and process-dependent modifications that can occur during apoptosis or necrosis. Inclusion of surface proteins should be emphasized. Non-protein antigens would also be required, such as lipids, carbohydrates, and other small molecules. Human-derived antigens represent a priority, but environmental antigen sources such as pathogens and viruses cannot be excluded. Technology is emerging that is approaching these goals through expressing the human genome (57) or virome (58) for the purpose of antibody screening (59). Continued development of these technologies will likely include tertiary structure and post-translational modifications that are important in the formation of many epitopes. Particular focus on MS antigens should start with well-characterized CNS tissue and CSF from early and progressive disease. Now that links between the CNS, CSF, cervical lymphnodes, and peripheral B cells are better understood (19, 60–62), the isolation and examination of particular B lineage subsets in the circulation will be of value. Next generation B cell antibody sequencing now allows comprehensive sequencing of B cell populations to create a repertoire that can be used to guide selection of clones for antigen screening. This can now be coupled with single cell approaches to pair the native VH and VL. The use of animal models to test and validate the contribution of MS-derived immunoglobulin to pathology should be leveraged. Finally, large scale, multi-center studies involving a number of investigators are best suited to tackle this expensive and high-risk endeavor.

## Author Contributions

The original hypothesis was conceived by DH and KO who also co-directed the project. The study was initiated and designed by DH and KO. The experiments were designed and data were collected by KO, SW, and DH. Data were analyzed and interpreted by SC, PS, AC, KO and SW. SW, SC, PS, and KO wrote the manuscript.

## Conflict of Interest Statement

Kevin C. O’Connor received speaking fees from EMD Serono and consults for Scitemex. The other co-authors declare that the research was conducted in the absence of any commercial or financial relationships that could be construed as a potential conflict of interest.
