# Supplementary material for: Investigating the Antigen Specificity of Multiple Sclerosis Central Nervous System-Derived Immunoglobulins
Source: Front Immunol. 2015 Nov 25;6:600. doi: 10.3389/fimmu.2015.00600 (PMC4663633; doi:10.3389/fimmu.2015.00600)
Supplement: Supplementary file 1 [file image_1.pdf]

Supplementary Figure 1. MS and control-derived rIgG binding to CNS cells and MOG-transfected cells detected with a cell-based assay. Binding to extracellular antigens present on the surface of the CNS-derived cell lines, human oligogendroglioma cell line (HOG), a human neuroblastoma cell line (SKNSH) or to a control non-CNS cell line (293T) is shown in the 1st, 2nd and 3rd columns respectively. Binding to Jurkat cells transfected with MOG-GFP or GFP alone is shown in the 4th and 5th columns respectively. Antibody binding was detected using anti-human secondary antibodies and is shown with blue histograms. Background binding observed with secondary antibody alone is shown in red.

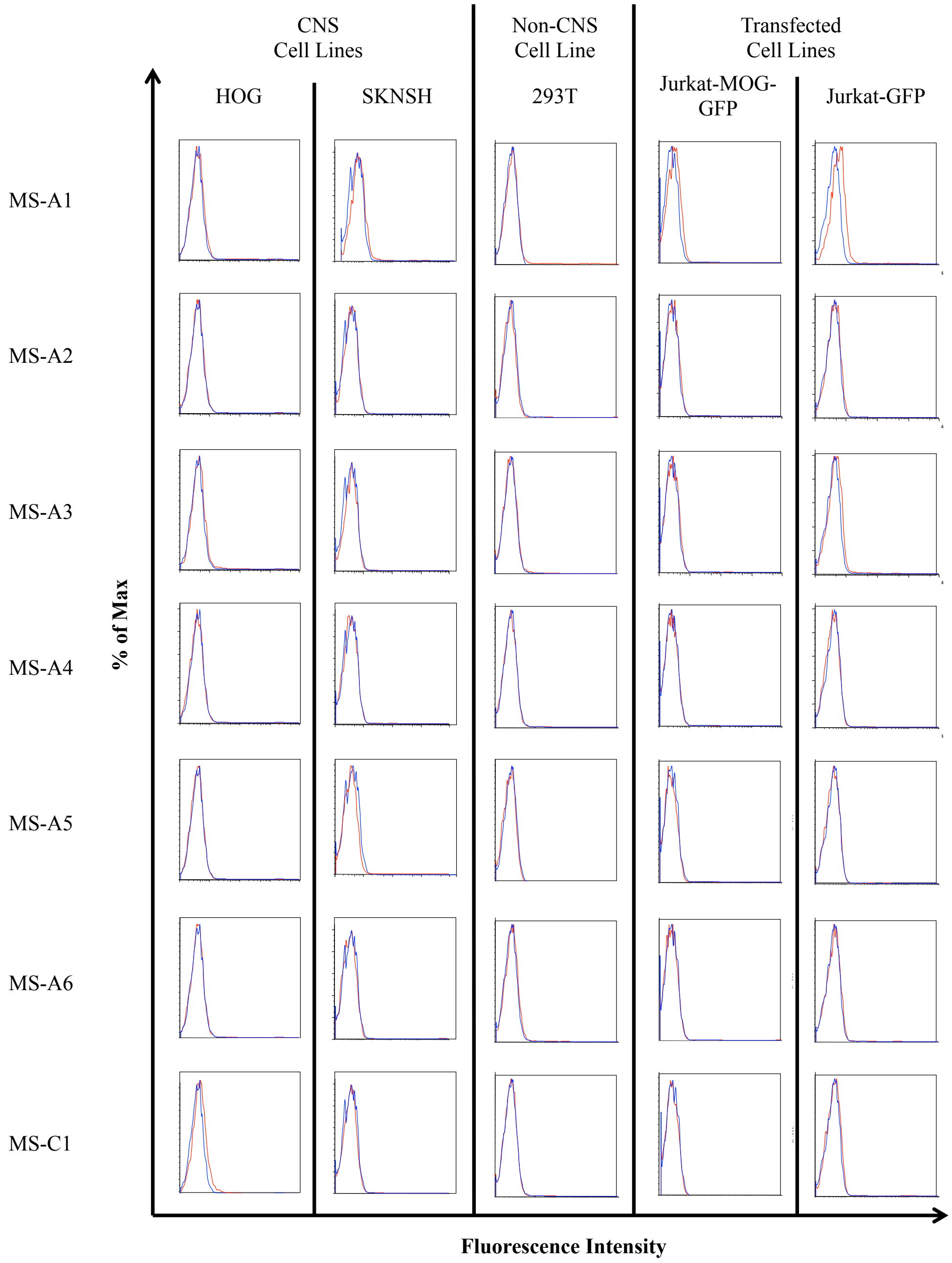

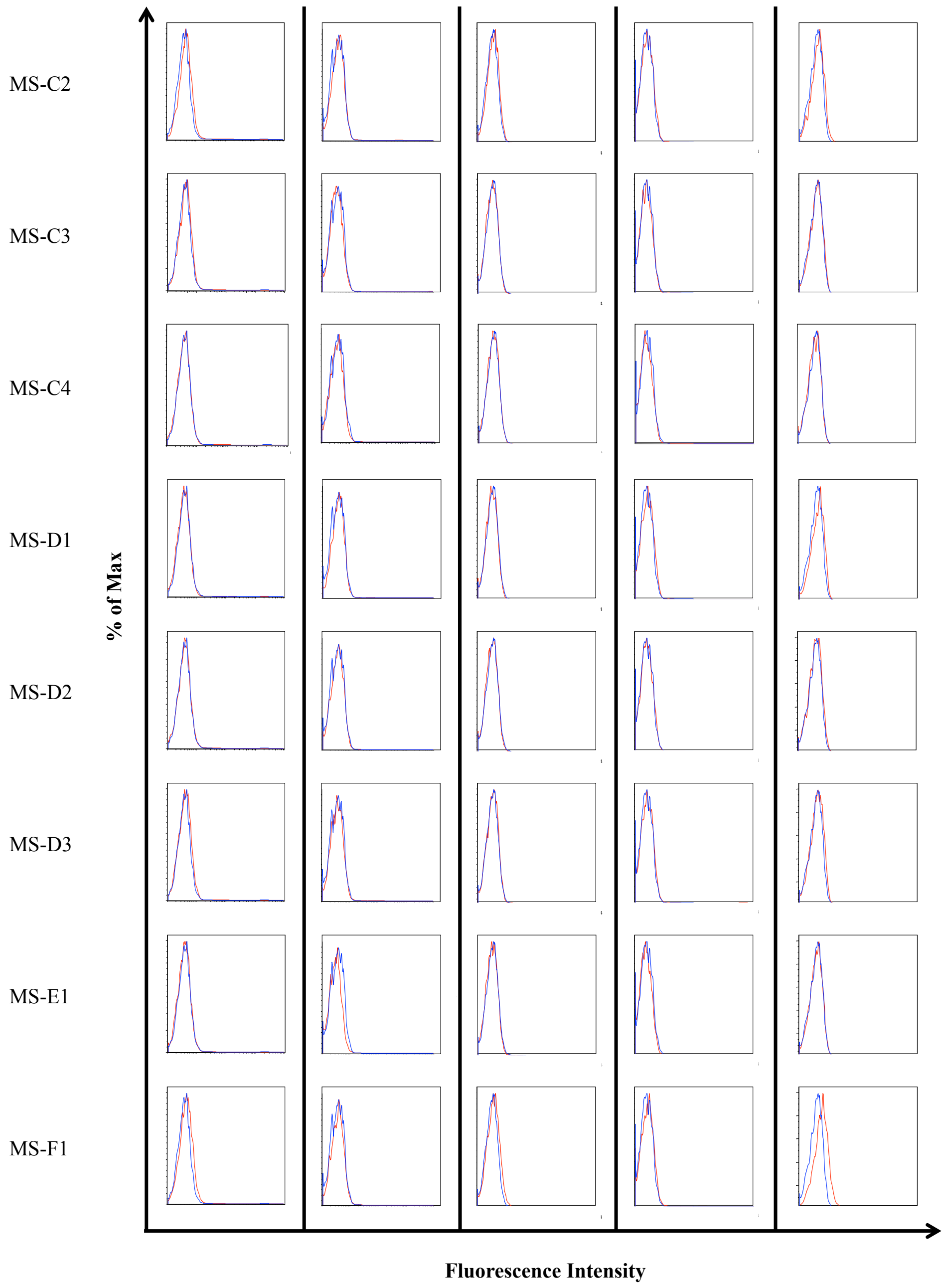
MS-C2
MS-C3
MS-C4
MS-D1
MS-D2
MS-D3
MS-E1
MS-F1
% of Max
Fluorescence Intensity

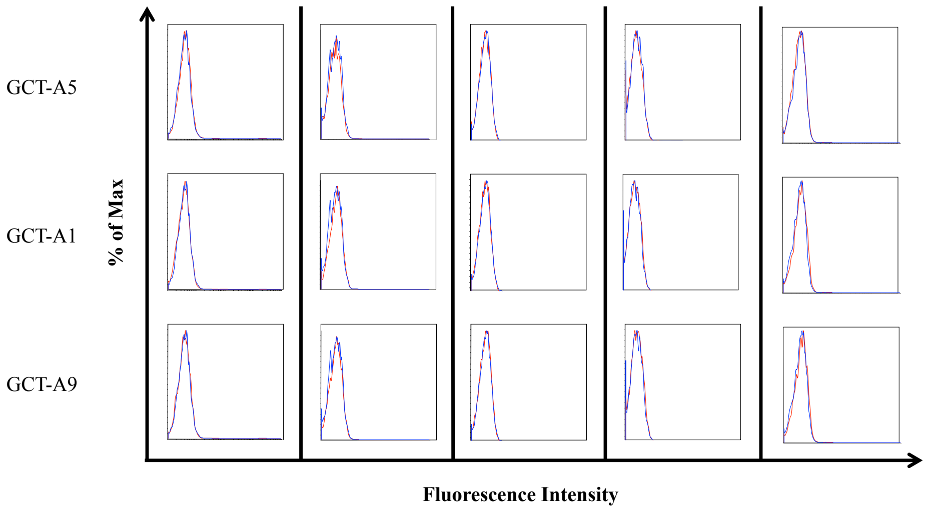
GCT-A5
GCT-A1
GCT-A9
% of Max
Fluorescence Intensity
