# Supplementary material for: Investigating the Antigen Specificity of Multiple Sclerosis Central Nervous System-Derived Immunoglobulins
Source: Front Immunol. 2015 Nov 25;6:600. doi: 10.3389/fimmu.2015.00600 (PMC4663633; doi:10.3389/fimmu.2015.00600)
Supplement: Supplementary file 2 [file image_2.pdf]

Supplementary Figure 2. ProtoArray-derived data show targets recognized by rIgG derived from B cells residing in the CNS of patients with multiple sclerosis. Reactivity of MS derived rIgG was compared to that of rIgG derived from controls. Z-scores that were larger than 3 in the MS cohort and did not reach this threshold in any of the control arrays are shown and highlighted in red. The rIgGs from both the MS and controls groups were pooled (up to 3 rIgG per array) during the initial scouting to maximize efficient use of the arrays. MS array 1 included MS-A1, A4 and A6; MS array 2 included MS-C1, C2 and C4; MS array 3 included MS-F1, MS-B1 and MS-D1. An equal number of control arrays were also run side-by-side with the MS samples. Control array 1 included GCT-A4, A5 and A6; control array 2 included IBM-A1, A2 and A3 and control array 3 included IBM-B1, A2 and A3.

MS array 1	MS array 2	MS array 3	Control array 1	Control array 2	Control array 3	Database ID	Description
MS-derived rIgG			Control-derived rIgG				
MS-A1, A4 and A6	MS-C1, C2 and C4	MS-F1, MS-B1 and MS-D1	GCT-A4, A5 and A6	IBM-A1, A2 and A3	IBM-B1, A2 and A3		
18.72289	-0.27933	0.14805	-0.15959	-0.22101	-0.1595	BC002859.1	Zinc finger protein 434 (ZNF434)
9.28843	-0.26154	-0.24613	-0.12188	-0.03497	-0.05196	NM_144582.1	Protein TEX261
5.14139	1.04615	4.67008	1.69579	1.50156	1.59497	BC064512.1	Ubiquitin protein ligase E3 component n-recognin 2 (UBR2)
3.93412	-0.14934	-0.21126	-0.10147	0.14758	0.03775	BC021282.1	Zinc finger protein 444
3.84769	0.39126	-0.11347	0.93392	2.81065	0.51889	NM_032138.2	Kelch repeat and BTB (POZ) domain containing 7 (KBTBD7)
2.49117	9.29009	3.15964	0.76089	0.79793	1.01824	NM_080664.1	Chromosome 14 open reading frame 126 (C14orf126)
0.08156	5.52918	-0.1927	0.28658	0.42184	0.33986	NM_012148.1	Double homeobox, 3 (DUX3)
0.30002	4.47831	3.22296	0.07619	0.42166	-0.0457	NM_004766.1	Coatomer protein complex, subunit beta 2 (beta prime) (COPB2)
0.23197	3.7005	-0.26932	-0.05105	0.36803	0.09027	NM_017871.3	Cleavage and polyadenylation specific factor 3-like (CPSF3L)
0.65573	3.62779	4.02304	0.02715	0.40577	0.58791	NM_032403.1	Protocadherin gamma subfamily C, 3 (PCDHGC3), transcript variant 3
0.22647	3.21718	2.74065	-0.06065	-0.05855	0.03679	NM_015138.2	Rtf1, Paf1/RNA polymerase II complex component, homolog (S. cerevisiae) (RTF1)
2.07074	1.50829	6.98401	0.77787	1.00109	2.57242	BC063390.1	Hypothetical protein LOC51233, mRNA (cDNA clone MGC:75009 IMAGE:5170001), complete cds.
2.7561	0.96952	5.95064	0.62834	2.70619	0.60574	BC010629.1	Outer dense fiber of sperm tails 2 (ODF2)
0.47418	2.29552	5.49717	0.11357	0.3455	0.47185	NM_024038.2	Chromosome 19 open reading frame 43 (C19orf43)
0.56177	2.57615	5.28546	0.03212	0.07246	0.79862	BC040946.1	CWC15 homolog (S. cerevisiae) (HSPC148)
1.01722	1.25852	5.19654	0.06161	0.0742	1.6346	BC033817.1	PC4 and SFRS1 interacting protein 1 (PSIP1)
0.12629	0.33634	5.18646	-0.02773	-0.05489	0.46749	BC007346.2	Uncharacterized protein C16orf14
1.03445	2.49141	5.11891	0.17941	0.27126	1.93482	NM_024790.2	Centrosome and spindle pole associated protein 1 (CSPP1), transcript variant 2
0.35112	-0.00418	4.61786	0.05543	0.233	0.82309	NM_206855.1	Quaking homolog, KH domain RNA binding (mouse) (QKI), transcript variant 4
0.37979	1.94793	4.59748	0.39256	0.36401	0.34195	BC002914.1	WAS/WASL-interacting protein family member 1
0.46057	-0.07768	4.49425	0.05389	0.23841	0.71821	NM_175609.1	ADP-ribosylation factor GTPase activating protein 1 (ARFGAP1), transcript variant 2
0.06707	0.17261	4.48135	-0.1073	0.01918	0.20388	BC066353.1	LIM homeobox transcription factor 1, alpha (LMX1A)
0.55453	-0.01256	4.47046	0.06213	0.30619	0.66681	NM_017612.1	Zinc finger CCHC domain-containing protein 8
1.15534	0.48698	4.4628	0.13209	0.18495	0.78041	BC058861.1	Sulfotransferase family, cytosolic, 1C, member 4 (SULT1C4)
1.46573	0.93892	4.23939	1.9374	1.53527	2.42601	NM_014763.2	Mitochondrial ribosomal protein L19 (MRPL19), nuclear gene encoding mitochondrial protein
-0.23435	-0.32902	4.23314	-0.18617	-0.26433	-0.20008	NM_005659.1	Ubiquitin fusion degradation 1 like (yeast) (UFD1L), transcript variant 1, mRNA.
-0.09652	0.59499	4.18797	-0.11552	-0.11987	-0.08894	BC021670.1	Ferritin, light polypeptide (FTL)
0.6301	0.54478	4.14361	1.47612	0.81662	0.81228	NM_006790.1	Myotilin (MYOT)
0.09096	0.24061	4.05409	0.21989	0.11963	0.25887	NM_032349.1	Nudix (nucleoside diphosphate linked moiety X)-type motif 16-like 1 (NUDT16L1)
-0.07669	0.0489	4.04481	-0.05346	0.13237	0.01461	BC093864.1	Disks large homolog 3
0.28974	1.18816	3.99944	0.49579	0.43318	0.5919	BC007363.1	Branched chain ketoacid dehydrogenase kinase (BCKDK)
0.25977	1.01451	3.95669	0.1242	0.21728	1.02487	XM_379498.2	PREDICTED: Homo sapiens hypothetical LOC401363 (LOC401363)
0.68627	0.20687	3.86878	-0.00647	0.9831	0.21279	BC091500.1	Outer dense fiber protein 2
0.00801	0.10017	3.76535	-0.11089	-0.06047	0.35882	NM_001551.1	Immunoglobulin (CD79A) binding protein 1 (IGBP1)
0.08402	0.69072	3.68127	-0.09272	-0.05278	0.23479	NM_021178.1	Cyclin B1 interacting protein 1 (CCNB1IP1), transcript variant 1
0.43683	2.35463	3.6605	0.37833	0.52926	1.02677	BC020726.1	Sciellin (SCEL)
-0.10926	0.41951	3.5986	-0.10661	0.37606	-0.04476	NM_156036.1	Homeobox protein Hox-B6
1.68869	0.47966	3.51412	0.48139	1.79311	1.01539	BC005055.1	Forkhead box P1 (FOXP1)
0.76532	-0.04473	3.34313	0.05167	0.31335	1.14891	BC007811.2	Hypothetical protein FLJ22795 (FLJ22795)
0.15959	-0.02956	3.22558	-0.08844	-0.0437	0.20521	NM_000190.2	Hydroxymethylbilane synthase (HMBS), transcript variant 1
0.23733	0.17706	3.20118	-0.02636	0.15666	0.68351	BC010155.2	Adrenodoxin-like protein, mitochondrial
0.51501	1.51221	3.19493	0.09625	0.31615	0.75994	NM_001009880.	Chromosome 22 open reading frame 9 (C22orf9), transcript variant 2
0.90531	0.44017	3.12536	0.25006	0.68579	0.92664	NM_001819.1	Chromogranin B (secretogranin 1) (CHGB)
