# Supplementary material for: Investigating the Antigen Specificity of Multiple Sclerosis Central Nervous System-Derived Immunoglobulins
Source: Front Immunol. 2015 Nov 25;6:600. doi: 10.3389/fimmu.2015.00600 (PMC4663633; doi:10.3389/fimmu.2015.00600)

Supplementary Figure 3. MS and control-derived rIgG binding to permeabilized a human oligodendrocyte cells. Binding of MS (A) or control-derived (B) rIgG to a human oligodendrocyte cell line (HOG) to evaluate intracellular binding. The blue histograms show secondary antibody alone and the red show rIgG with the addition of the secondary antibody.

A.

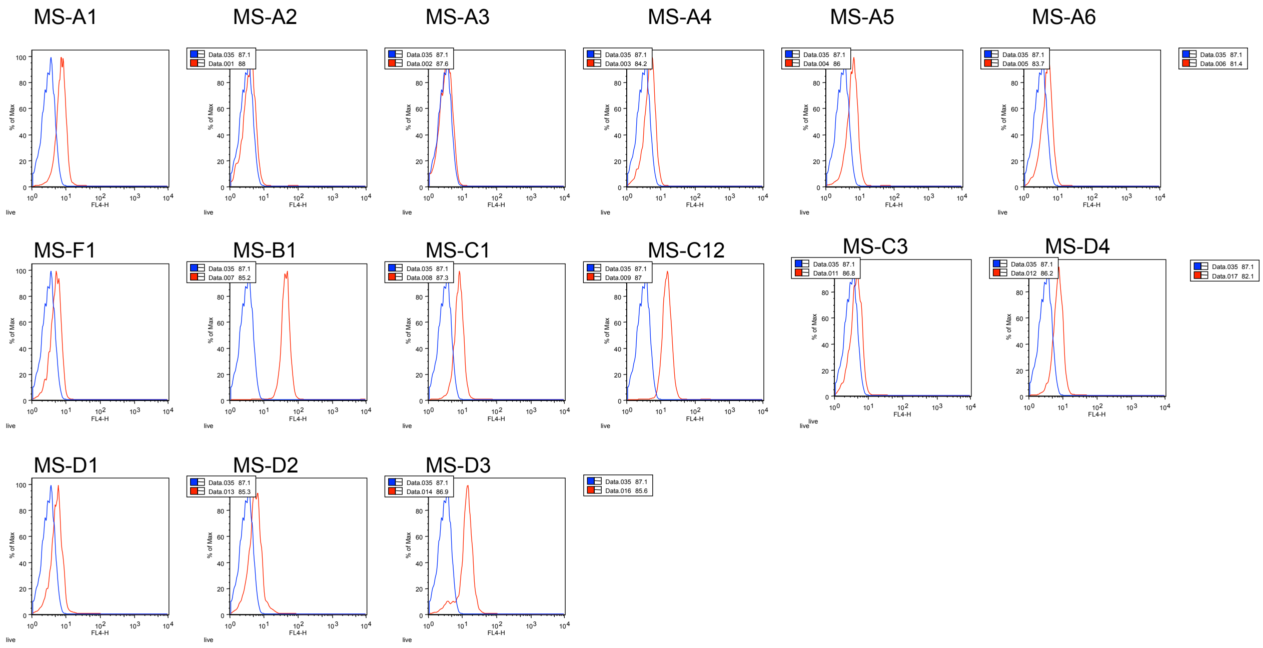

B.

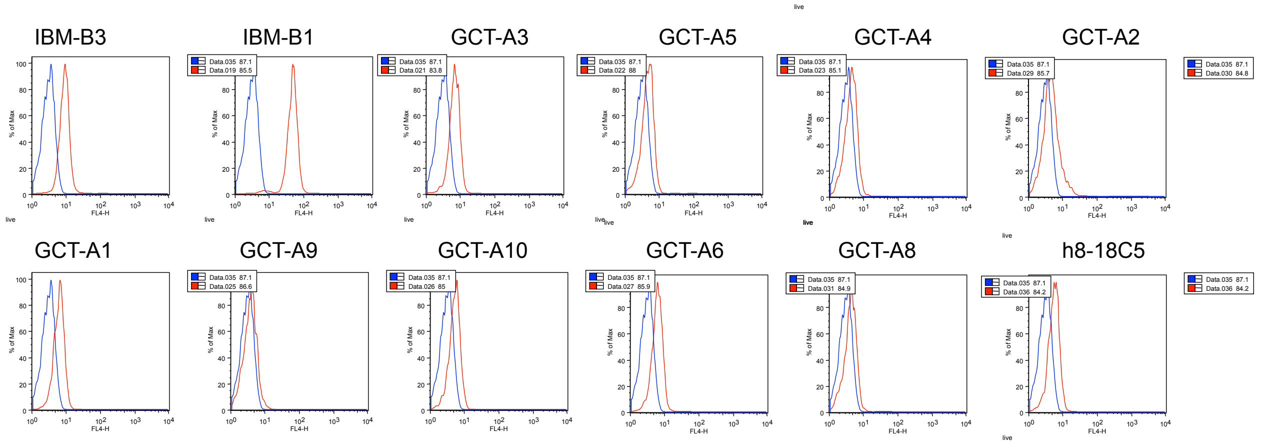

Supplement: Supplementary file 3 [file image_3.pdf]
