# Supplementary material for: Investigating the Antigen Specificity of Multiple Sclerosis Central Nervous System-Derived Immunoglobulins
Source: Front Immunol. 2015 Nov 25;6:600. doi: 10.3389/fimmu.2015.00600 (PMC4663633; doi:10.3389/fimmu.2015.00600)
Supplement: Supplementary file 4 [file table_1.pdf]

Supplementary Table 1. Recombinant immunoglobulins used in this study

Case	rIgG	Tissue source	Tissue characteristics
MS-A	MS-A1	MS lesion	White matter
	MS-A2	MS lesion	White matter
	MS-A3	MS lesion	White matter
	MS-A4	MS lesion	White matter
	MS-A5	MS lesion	White matter
	MS-A6	MS lesion	White matter
MS-B	MS-B1	MS lesion	White matter
MS-C	MS-C1	MS lesion	White matter
	MS-C2	MS lesion	White matter
	MS-C3	MS lesion	White matter
	MS-C4	MS lesion	White matter
MS-D	MS-D1	MS lesion	White matter
	MS-D2	MS lesion	White matter
	MS-D3	MS lesion	White matter
	MS-D4	MS lesion	White matter
MS-E	MS-E1	MS lesion	White matter
MS-F	MS-F1	MS CNS meninges	Meningeal follicle
GCT-A	GCT-A1	Intracranial germinoma	tumor infiltrating lymphocytes
	GCT-A2	Intracranial germinoma	tumor infiltrating lymphocytes
	GCT-A3	Intracranial germinoma	tumor infiltrating lymphocytes
	GCT-A4	Intracranial germinoma	tumor infiltrating lymphocytes
	GCT-A5	Intracranial germinoma	tumor infiltrating lymphocytes
	GCT-A6	Intracranial germinoma	tumor infiltrating lymphocytes
	GCT-A7	Intracranial germinoma	tumor infiltrating lymphocytes
	GCT-A8	Intracranial germinoma	tumor infiltrating lymphocytes
	GCT-A9	Intracranial germinoma	tumor infiltrating lymphocytes

	GCT-A10	Intracranial germinoma	tumor infiltrating lymphocytes
IBM-A	IBM-A1	IBM muscle tissue	biopsy
	IBM-A2	IBM muscle tissue	biopsy
	IBM-A3	IBM muscle tissue	biopsy
IBM-B	IBM-B1	IBM muscle tissue	biopsy
	IBM-B2	IBM muscle tissue	biopsy
	IBM-B3	IBM muscle tissue	biopsy
mAb	h8-18C5	humanized Anti-MOG mAb	Humanized 8-18C5
