# Supplementary material for: Investigating the Antigen Specificity of Multiple Sclerosis Central Nervous System-Derived Immunoglobulins
Source: Front Immunol. 2015 Nov 25;6:600. doi: 10.3389/fimmu.2015.00600 (PMC4663633; doi:10.3389/fimmu.2015.00600)
Supplement: Supplementary file 5 [file table_2.pdf]

# Supplementary Table 2. Sequence characteristics of immunoglobulin variable regions used in this study

| | HEAVY | | | | | LIGHT | | | |
|---|---|---|---|---|---|---|---|---|---|
| **ID** | **VH** | **D** | **JH** | **CDR3* (aa)** | **Mutations** (aa)** | **Vk** | **Jk** | **CDR3* (aa)** | **Mutations** (aa)** |
| MS-A1 | IGHV3-15 | IGHD5-12 | IGHJ4 | CTTDD**T**G**H**DWGHW | 7 | IGKV1-12 | IGKJ1 | CQQANSFPSF | 3 |
| MS-A2 | IGHV4-59 | IGHD1-26 | IGHJ5 | CARG**K**DW**L**D**P**W | 8 | IGKV1-9 | IGKJ2 | CQQ**V**N**A**YPYTF | 3 |
| MS-A3 | IGHV6-1 | IGHD6-6 | IGHJ4 | CA**T**GGQY**T**S**W**SFD**S**W | 9 | IGKV1-16 | IGKJ4 | CQQY**HNL**LTF | 8 |
| MS-A4 | IGHV3-30 | IGHD2-2 | IGHJ4 | CA**K**EIDL**A**YLN**S**W | 6 | IGKV1-9 | IGKJ2 | CQQ**V**N**A**YPYTF | 3 |
| MS-A5 | IGHV3-30 | IGHD2-2 | IGHJ4 | CA**K**EIDL**A**YL**NS**W | 7 | IGKV1-9 | IGKJ2 | CQQ**V**N**A**YPYTF | 3 |
| MS-A6 | IGHV1-18 | IGHD3-10 | IGHJ4 | C**V**RGVV**A**GGDDYW | 9 | IGKV1-9 | IGKJ2 | CQQ**V**N**A**YPYTF | 3 |
| MS-B1 | IGHV2-26 | IGHD3-22 | IGHJ6 | CAR**L**HPVAYEGYY**H**HVHG**V**DVW | 7 | IGKV1-39 | IGKJ5 | C**L**QSY**N**TPLTF | 5 |
| MS-C1 | IGHV3-9 | IGHD5-12 | IGHJ4 | CARDSGYEFLDYW | 12 | IGKV1-33 | IGKJ4 | CQQYDNL**R**PLTF | 3 |
| MS-C2 | IGHV2-5 | IGHD1-26 | IGHJ6 | CAHTRIVGATRHSYYH**A**MDVW | 9 | IGKV1-39 | IGKJ1 | CQQSYTFSWTF | 8 |
| MS-C3 | IGHV2-5 | IGHD2-2 | IGHJ4 | CAHVFRDCS**T**T**R**CYDRGYDYW | 8 | IGKV1-33 | IGKJ4 | CQ**H**YD**S**LPLTF | 9 |
| MS-C4 | IGHV1-2 | IGHD6-25 | IGHJ3 | CARDGGRH**T**SGYYEWTA**L**DIW | 10 | IGKV1-39 | IGKJ1 | CQQSYTFSWTF | 8 |
| MS-D1 | IGHV1-46 | IGHD5-18 | IGHJ3 | CTTELGYS**L**GLRA**S**DVW | 8 | IGKV3-11 | IGKJ2 | C**HL**R**G**NWPRTF | 5 |
| MS-D2 | IGHV1-46 | IGHD5-18 | IGHJ3 | CTTELGYS**L**GLRA**S**DVW | 8 | IGKV1-39 | IGKJ2 | CQQS**D**S**S**PYTF | 6 |
| MS-D3 | IGHV3-7 | IGHD2-2 | IGHJ6 | CAREVR**D**CSS**ST**CYAEDLIY**F**YYYYMDVW | 9 | IGKV3-11 | IGKJ2 | C**HL**R**G**NWPRTF | 5 |
| MS-D4 | IGHV5-51 | IGHD5-12 | IGHJ6 | CARHSPPGYDYNAMDVW | 6 | IGHKV3-11 | IGKJ2 | C**HL**R**G**NWPRTF | 5 |
| MS-E1 | IGHV2-5 | IGHD1-14 | IGHJ4 | CAHRPIIEHNRIPFDFW | 4 | IGKV1-39 | IGKJ1 | CQQSYSTLWTF | 4 |

| | | | | | | | | | |
|---|---|---|---|---|---|---|---|---|---|
| MS-F1 | IGHV3-23 | IGHD3-16 | IGHJ6 | CANARFYCSTSNCPAAGMDVW | 11 | IGKV2-28 | IGKJ4 | CMQSLQTPPTF | 6 |
| | | | | | | | | | |
| GCT-A1 | IGHV4-61 | IGHD3-22 | IGHJ3 | CARDLLYYSDSSGFYGAFDIW | 3 | IGKV4-1 | IGKJ1 | CQQYYTTLGTF | 1 |
| GCT-A2 | IGHV3-43 | IGHD2-8 | IGHJ4 | CAKDSSRDILLGTTTGFMDVW | 4 | IGKV1-33 | IGKJ4 | CQQYDNLPPTF | 3 |
| GCT-A3 | IGHV4-61 | IGHD2-2 | IGHJ3 | CARGLVTYCRTTNCYDVFDIW | 5 | IGKV1-12 | IGKJ2 | CQQANSFPYTF | 5 |
| GCT-A4 | IGHV3-7 | IGHD6-19 | IGHJ6 | CARWAHSPVAESFHYYYYAMDVW | 2 | IGKV3-20 | IGKJ2 | CQQYGNSPPYTF | 6 |
| GCT-A5 | IGHV5-10 | NI | IGHJ6 | CARDYGDVW | 9 | IGKV4-1 | IGKJ1 | CQQYYTIPRTF | 2 |
| GCT-A6 | IGHV3-33 | IGHD3-3 | IGHJ4 | CARDSTPFNYDFWSGYYSGVPPTPATADYW | 2 | IGKV1-33 | IGKJ5 | CQQYGDLPRAF | 5 |
| GCT-A7 | IGHV5-51 | IGHD3-22 | IGHJ4 | CARPSSMMSPFDYW | 6 | IGKV1-33 | IGKJ5 | CQQYGDLPRAF | 4 |
| GCT-A8 | IGHV5-10 | IGHD2-2 | IGHJ4 | CARHDGAAMARYGYW | 4 | IGKV2-30 | IGKJ1 | CMQGTQWPWTF | 4 |
| GCT-A9 | IGHV1-24 | IGHD3-22 | IGHJ5 | CATASRDYYDSSGYLSTKNWFDPW | 0 | IGKV1-33 | IGKJ4 | CQQYDDLPLTF | 3 |
| GCT-A10 | IGHV4-61 | IGHD3-22 | IGHJ3 | CARDLVYYSDSSGFYGAFDIW | 4 | IGKV4-1 | IGKJ1 | CQQYYTTLGTF | 1 |
| | | | | | | | | | |
| IBM-A1 | IGHV3-9 | IGHD3-22 | IGHJ4 | CVKEDYGTGGYYRFDHW | 10 | IGKV1-39 | IGKJ2 | CQQSYTSPYTF | 5 |
| IBM-A2 | IGHV2-70 | IGHD3-10 | IGHJ6 | CARVRVVRGAGYYYGMDVW | 11 | IGKV4-1 | IGKJ4 | CQQYYDTPLTF | 11 |
| IBM-A3 | IGHV5-51 | IGHD3-3 | IGHJ6 | CARLKYHFWNGHDYYYYGMDVW | 13 | IGKV4-1 | IGKJ2 | CQQYYSPPYTF | 4 |
| IBM-B1 | IGHV1-69 | IGHD1-26 | IGHJ1 | CEGGFQHW | 27 | IGKV2-28 | IGKJ2 | CMQARQTVYTF | 8 |
| IBM-B2 | IGHV3-9 | IGHD3-10 | IGHJ4 | CVKDRGYASLSFGDLEYW | 5 | IGKV3-15 | IGKJ4 | CQQYDNWPSF | 7 |
| IBM-B3 | IGHV1-2 | IGHD3-3 | IGHJ4 | CASLRIKIFGLIINPYDYW | 12 | IGKV3-20 | IGKJ5 | CQQYANSVF | 7 |
| | | | | | | | | | |
| H8-18C5 | IGHV1-18 | IGHD4-23 | IGHJ1 | CATGNTMVNMPYW | 10 | IGKV4-1 | IGKJ4 | CQNDHSYPLTF | 1 |

* Bold amino acids indicate replacement mutations

** $V_H$ and $V_K$ gene segment mutation count does not include those indicated in the CDR3

The sequences of the VH and VL domains listed in the table have been deposited in GenBank under accession numbers KT984419-KT984484 following the same order in which they appear in the table.

NI = not identifiable (usually due to particularly short D-segments)
