# Supplementary material for: Investigating the Antigen Specificity of Multiple Sclerosis Central Nervous System-Derived Immunoglobulins
Source: Front Immunol. 2015 Nov 25;6:600. doi: 10.3389/fimmu.2015.00600 (PMC4663633; doi:10.3389/fimmu.2015.00600)
Supplement: Supplementary file 6 [file table_3.pdf]

Supplemental Table 3. Plotted values of MS and control-derived rIgG binding to neurofilament light (NF-L) by solid phase ELISA shown in Figure 1.

Identification of rIgG	ΔAbs (455nm)
MS-A1	0.229
MS-D2	0.412
MS-A2	0.384
MS-A6	0.245
MS-A4	0.619
MS-A5	0.322
MS-B1	0.881
MS-F1	0.309
MS-C1	0.472
MS-C2	1.139
MS-C4	0.964
MS-C3	0.176
MS-D1	0.455
GCT-A8	0.050
GCT-A1	0.000
GCT-A10	0.226
GCT-A9	0.101
GCT-A3	0.000
GCT-A2	0.154
GCT-A5	0.000
GCT-A6	0.879
