# Supplementary material for: Investigating the Antigen Specificity of Multiple Sclerosis Central Nervous System-Derived Immunoglobulins
Source: Front Immunol. 2015 Nov 25;6:600. doi: 10.3389/fimmu.2015.00600 (PMC4663633; doi:10.3389/fimmu.2015.00600)
Supplement: Supplementary file 7 [file table_4.pdf]

Supplemental Table 4. Plotted values of MS and control-derived rIgG binding to protocadherin gamma by solid phase ELISA shown in Figure 3.

Identification of rIgG	Abs (455nm)
MS-A1	0.207
MS-D2	0.036
MS-A2	0.072
MS-A6	0.010
MS-A4	0.040
MS-A5	0.045
MS-B1	0.135
MS-F1	0.048
MS-C2	0.710
MS-C3	0.019
MS-D1	0.186
IBM-A2	0.121
IBM-A3	0.032
IBM-A1	0.001
GCT-A10	1.161
GCT-A9	0.000
GCT-A5	0.016
GCT-A6	0.069
GCT-A2	0.046
GCT-A1	0.021
GCT-A3	0.093
Commercial anti-protocadherin	1.078
